# The Stellate Ganglion Nerve Block—A Novel Use for Electrical Storm in the Emergency Department: A Case Report

**DOI:** 10.5811/cpcem.62964

**Published:** 2026-09-08

**Authors:** Allison Smith, Louisa Weindruch, Rebecca Mayo, Jonathan Brewer

**Affiliations:** Baylor University Medical Center, Department of Emergency Medicine, Dallas, Texas

**Keywords:** electrical storm, regional anesthesia, ventricular tachycardia, stellate ganglion block, case report

## Abstract

**Introduction:**

Electrical storm is an emergent medical condition in which a patient experiences recurrent episodes of ventricular tachycardia or ventricular fibrillation. Traditional management includes intravenous antiarrhythmics and/or electrical cardioversion. However, in some cases, cardiac arrhythmias may persist despite these interventions. In rare circumstances, traditional therapies in the emergency department (ED) may prove to be futile. In such cases, a stellate ganglion nerve block (SGNB) can be considered.

**Case Report:**

We present a novel case in which a SGNB was performed in the ED on an awake patient experiencing recurrent cardioversions from his implantable cardioverter defibrillator shocks for ventricular tachycardia.

**Conclusion:**

In the setting of refractory ventricular dysrhythmias, sympathetic blockade of the left stellate ganglion can be a useful adjunct to conventional management in the ED.

## INTRODUCTION

Electrical storm is defined as three or more recurrent episodes of a malignant cardiac arrhythmia such as ventricular tachycardia (VT) or ventricular fibrillation (VF) in a 24-hour period. The etiology is varied, but it is theorized that sympathetic activation lowers the threshold for these malignant arrhythmias.^1^ Initial treatment in hemodynamically stable patients is intravenous (IV) antiarrhythmic drugs. If the patient is hemodynamically unstable or refractory to antiarrhythmics, cardioversion is the treatment of choice. When a patient is refractory to both medications and cardioversion, a stellate ganglion nerve block (SGNB) can be considered. The SGNB is postulated to function by temporarily blocking the cervical sympathetic chain.^2^ It has been largely used in the inpatient setting by cardiologists and anesthesiologists. However, in recent years, there has been emerging evidence on its utility in the emergency department (ED) for patients in cardiac arrest. These data are still limited and exist mostly as case reports and case series on patients in cardiac arrest. In this case, we present the novel use of the nerve block in the ED for treatment of refractory VT and electrical storm in an awake and hemodynamically stable patient with recurrent cardioversions from his automated implantable cardioverter defibrillator (AICD). To date, our case marks the first of its kind reported within the United States.

## CASE REPORT

A 60-year-old male with an extensive past medical history including end-stage renal disease, prior cerebrovascular accident, hypertension, recurrent pericardial effusions status post pericardial window, VT/VF arrest status post AICD placement, coronary artery disease status post coronary artery bypass graft, and heart failure with reduced ejection fraction of 30–35% who presented to the ED after his AICD delivered two episodes of cardioversion that day. The patient was well-appearing and had the following initial vital signs: blood pressure, 181/107 mm Hg; pulse, 82 beats/min (bpm); respiratory rate, 22 breaths/min; oxygenation,95% on room air; and temperature, 98.5 ºF. He was placed on continuous cardiac monitoring and an electrocardiogram initially demonstrated sinus tachycardia at 107 bpm. Intravenous access was established. A chest radiograph showed cardiomegaly without other acute abnormalities.

While the laboratory evaluation was pending, an episode of VT was noted on the telemetry monitor and coincided with the patient experiencing two more episodes of synchronized cardioversion from his AICD. His vital signs remained unchanged. He continued to have episodes of monomorphic VT and was witnessed to have nine additional appropriate synchronized cardioversions from his AICD within a matter of minutes, causing him significant pain. Medical management was attempted with IV amiodarone (150 mg) followed by a magnesium infusion (2 g). However, the patient continued to have recurrent episodes of VT with appropriate cardioversion and overdrive pacing attempts by his AICD. Given the failed pharmacologic interventions and patient’s extreme discomfort, SGNB was considered. The risks and benefits of the procedure as well as other possible interventions were discussed with the patient, and he elected to proceed with SGNB.

The patient was positioned to sit upright with his neck in slight extension and rotated to the right, contralateral to the side of the planned procedure. His left anterolateral neck was prepped and draped in sterile fashion. The linear ultrasound probe was placed in transverse orientation at the level of the cricoid cartilage, which is approximately at the level of the sixth cervical vertebra (C6). While the stellate ganglion typically spans from the seventh cervical vertebra (C7) to the first thoracic vertebra (T1), the vertebral artery arises anterior to C7, making C6 a safer landmark for puncture.^3^ After moving slightly laterally and caudally, the C6 and C7 transverse processes were identified. This included identification of the anterior tubercle of C6, also known as the Chassaignac tubercle. The vasculature was visualized using color Doppler, including the internal carotid artery, vertebrobasilar arteries, and internal jugular vein.

*CPC-EM Capsule*

What do we already know about this clinical entity?

*Stellate ganglion nerve blocks (SGNB), traditionally used in the inpatient setting, now have growing evidence for utility in patients with cardiac arrest.*

What makes this presentation of disease reportable?

*We present the novel use of a SGNB for electrical storm in an awake and hemodynamically stable patient in the emergency department (ED).*

What is the major learning point?

*When a patient in electrical storm is refractory to both medications and cardioversion, a SGNB should be considered.*

How might this improve emergency medicine practice?

*The SGNB can be a useful adjunct in the management of refractory cardiac arrhythmias in the ED.*

An in-plane approach with a 22-gauge block B-Braun needle was performed. The needle was inserted laterally and advanced medially until the hyperechoic tip was visualized at the prevertebral fascia of the longus colli muscle, between the posterior aspect of the carotid artery and the Chassaignac tubercle. Five milliliters of 1% lidocaine without epinephrine were injected incrementally and were visualized elevating the prevertebral fascia, just below the stellate ganglion (Images 1 and 2, Figure). The needle was removed without complication. The patient developed miosis and ptosis within 5–10 minutes of the SGNB, suggesting effective coverage of the nerve region.^4^ There were no further shocks delivered by the AICD and no further episodes of VT while in the ED. The patient was then admitted to the cardiology service for further management. During his hospitalization, he had no additional episodes of VT. He declined any additional procedures and was discharged on oral amiodarone on hospital day 4.

## DISCUSSION

The stellate ganglion is a group of sympathetic nerve fibers in the anterior neck. It is formed by fusion of the inferior cervical ganglion with the first thoracic ganglion at the level of C6 and C7.^5^ While it can be found bilaterally, it is believed that the left stellate ganglion plays a more significant role in autonomic regulation of the heart; hence, the left-sided blockade is generally performed.^5^ In the 1970s, researchers began to explore the connection between sympathetic activation and cardiac arrhythmias, with one of the first stellate ganglion blocks for antiarrhythmic purposes being reported in 1976.^4^,^6^ Since then, the technique has been used by cardiologists and anesthesiologists for management of arrhythmias refractory to traditional management. In recent years, cases have emerged revealing its use in the ED, particularly in the setting of cardiac arrest.^7^ Our case represents a novel use for the stellate ganglion block in the ED in a patient who was awake and hemodynamically stable.

Emergency physicians must be able to identify and quickly treat dysrhythmias, as recurrent ventricular arrhythmias can have a mortality rate up to 97%.^7^ Electrical storm is common in patients with AICDs, occurring in 5% of those placed for primary prevention and 25% of those placed for secondary prevention.^4^ Recent literature has highlighted SGNB as a successful antiarrhythmic in electrical storm. In a meta-analysis of 15 studies with over 500 patients in electrical storm, 70% had complete resolution of their ventricular arrhythmias after SGNB.^8^ In a prospective study of 115 patients, 92.2% had a reduction of at least 50% in the number of treated arrhythmic episodes following SGNB.^4^ Complications are rare, the most common being transient hoarseness and lightheadedness.^9^

The benefits of the SGNB in our case are multifactorial. Not only did the SGNB quickly relieve the patient’s severe discomfort from the repeated AICD shocks, it decreased the likelihood of hemodynamic instability and eliminated the need for further interventions in the ED. With the widespread use of ultrasound-guided procedures and the increasing prevalence of ultrasound-guided regional anesthesia, we propose that the SGNB is feasible for emergency physicians. Landmarks can easily be visualized on ultrasound, and the procedure requires minimal time and resources—the ideal scenario for a busy ED. Ultimately, this case demonstrates how the SGNB can be used as an adjunct for termination of recurrent ventricular arrhythmias in awake patients in the ED.

## CONCLUSION

When conventional measures fail for refractory ventricular dysrhythmias, left stellate ganglion sympathetic blockade is a useful emergency department adjunct.

## Figures and Tables

**Figure.**
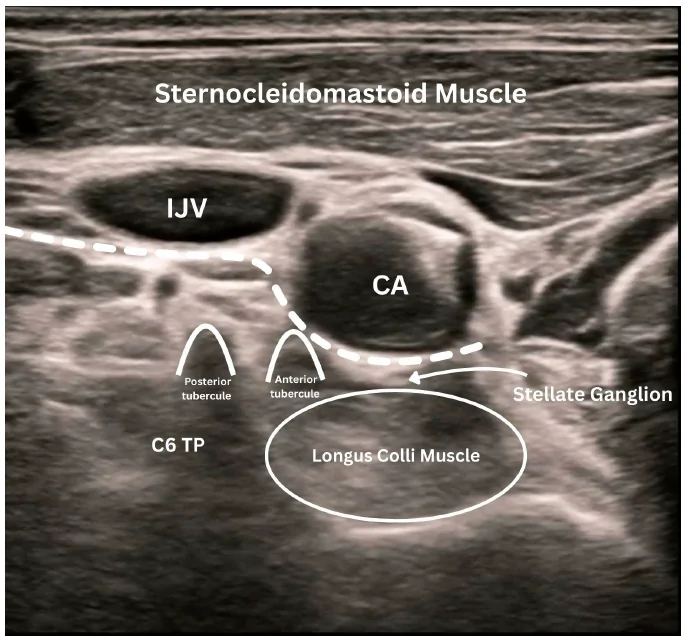
Diagram of neck anatomy. The star indicates stellate ganglion. *ASM*, anterior scalene muscle; *C6*, sixth cervical vertebra; *CA*, carotid artery; *IJV*, internal jugular vein; *LCM*, longus colli muscle; *PVF*, prevertebral fascia*; SCM*, sternocleidomastoid muscle; *VA*, vertebral artery.

**Image 1:**
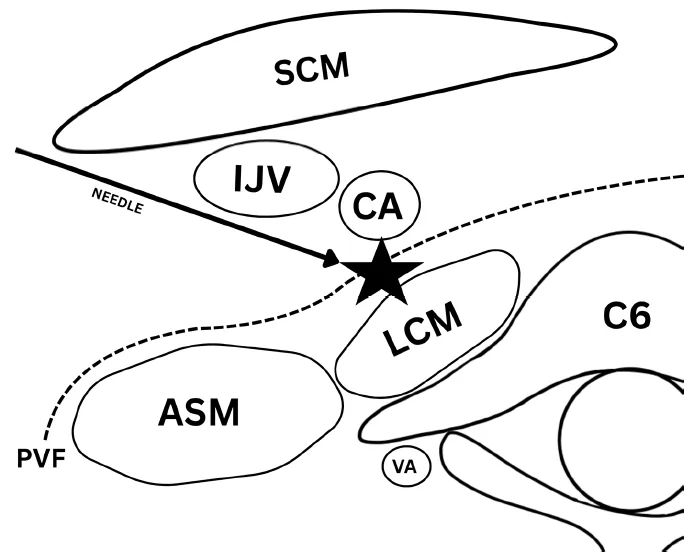
Ultrasound image of neck anatomy, with the dotted white line indicating prevertebral fascia. *C6*, sixth cervical vertebra; *CA*, carotid artery; *IJV*, internal jugular vein; *TP*, transverse process.

**Image 2:**
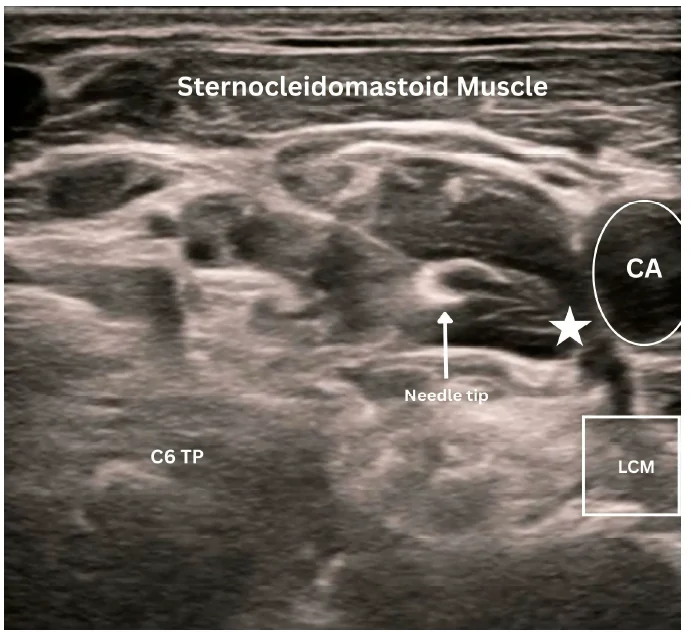
Ultrasound image of a needle injecting lidocaine. The star indicates lidocaine injectate traversing underneath the carotid artery; the white arrow points to the needle tip. *C6*, sixth cervical vertebra; *CA*, carotid artery; *LCM*, longus colli muscle; *TP*, transverse process.

## References

[1] Eifling M, Razavi M, Massumi A (2011). The evaluation and management of electrical storm. Tex Heart Inst J.

[2] Nademanee K, Taylor R, Bailey WE (2000). Treating electrical storm: sympathetic blockade versus advanced cardiac life support-guided therapy. Circulation.

[3] Oh D, Lee HS (2021). Atypical course of vertebral artery identified by ultrasound prescan before performing a stellate ganglion block. J Med Ultrasound.

[4] Savastano S, Baldi E, Compagnoni S (2024). Electrical storm treatment by percutaneous stellate ganglion block: the STAR study. Eur Heart J.

[5] Nair A, Bhoi S, Choudhary Y (2024). Cease the storm - successful stellate ganglion block in terminating refractory electrical storm. Am J Emerg Med.

[6] Grossman MA (1976). Cardiac arrhythmias in acute central nervous system disease. Successful management with stellate ganglion block. Arch Intern Med.

[7] Callipari C, Stone M, John D (2023). Intra-cardiac arrest use of stellate ganglion block for refractory ventricular tachycardia. J Emerg Med.

[8] Motazedian P, Quinn N, Wells GA (2024). Efficacy of stellate ganglion block in treatment of electrical storm: a systematic review and meta-analysis. Sci Rep.

[9] Goel V, Patwardhan AM, Ibrahim M (2019). Complications associated with stellate ganglion nerve block: a systematic review. Reg Anesth Pain Med.

[10] Margus C, Correa A, Cheung W (2020). Stellate ganglion nerve block by point-of-care ultrasonography for treatment of refractory infarction-induced ventricular fibrillation. Ann Emerg Med.

